# Oncology services supply in Colombia

**DOI:** 10.25100/cm.v49i1.3620

**Published:** 2018-03-30

**Authors:** Eliana Murcia, Jairo Aguilera, Carolina Wiesner, Constanza Pardo

**Affiliations:** 1 Grupo Evaluación y Seguimiento de Servicios Oncológicos, Instituto Nacional de Cancerología, Bogotá. D.C., Colombia.; 2 Dirección General, Instituto Nacional de Cancerología, Bogotá. D.C., Colombia.; 3 Grupo Vigilancia Epidemiológica del Cáncer, Instituto Nacional de Cancerología, Bogotá, D.C., Colombia.

**Keywords:** Health services, oncology service, hospital, cancer, Colombia, servicios de salud, servicio de oncología en hospital, cáncer, Colombia

## Abstract

**Objective:**

To characterize the current status of oncological services supply in Colombia.

**Methods:**

A descriptive analysis of oncological services for cancer care in the adult and infant population that meet the requirements for operation according to the Special Register of Health Service Providers was carried out. The case - by - provider ratio was calculated based on the cancer incidence estimated for Colombia by the National Cancer Institute.

**Results:**

Were identified 1,780 qualified oncology health services in the country related to specialties for providing care to cancer patients. Twenty five providers nationwide had all three qualified services: chemotherapy, radiotherapy and surgery. Nearly 50% of the offer was concentrated in Bogotá, Antioquia and Valle del Cauca. Putumayo and the Amazonas group departments, with the exception of Vaupés, did not show any oncological services. Healthcare Providers were responsible for 87.8%, and independent professionals provided 12.2%. Outpatient services were 66.7% of oncology services, 17.4% was diagnostic support services and therapeutic complementation, and 15.9% was surgical services. 87.9% of the oncological service offer in Colombia takes place in the private sector.

**Conclusions:**

The ratio between the service groups is asymmetric, with few providers jointly offering the basic services for oncology treatment, which reflects how provision is fragmented. It is necessary to redefine the concept of oncology service under a comprehensive care approach and the importance of enabling functional units, comprehensive treatment centers and other forms of care.

## Resumen

**Objetivo:**

Caracterizar la situación actual de la oferta de servicios oncológicos en Colombia.

**Métodos:**

Se realizó un análisis descriptivo de los servicios oncológicos para la atención de cáncer en población adulta e infantil, que cumplieron con los requisitos para su funcionamiento de acuerdo al Registro Especial de Prestadores de Servicios de Salud. La razón de casos por prestador se calculó a partir de la incidencia de cáncer estimada para Colombia por el Instituto Nacional de Cancerología.

**Resultados:**

Se identificaron 1,780 servicios de salud oncológicos habilitados en el país relacionados con especialidades para la atención de pacientes con cáncer. 25 prestadores a nivel nacional contaron con los tres servicios habilitados: quimioterapia, radioterapia y cirugía. Cerca del 50% de la oferta se concentró en Bogotá, Antioquia y Valle del Cauca. Los departamentos de Putumayo y del grupo Amazonas, con excepción de Vaupés, no registraron servicios oncológicos. El 87.8% fue ofertado por Instituciones Prestadoras de Salud y el 12.2% fue provisto por profesionales independientes. El 66.7% de los servicios oncológicos eran de consulta externa, el 17.4% eran servicios de apoyo diagnóstico y complementación terapéutica y el 15.9% servicios quirúrgicos. El 87.9% de la oferta de servicios oncológicos en Colombia está en el sector privado.

**Conclusiones:**

La relación entre los grupos de servicios es asimétrica, con pocos prestadores que ofertan de forma conjunta los servicios bases del tratamiento oncológico, lo cual refleja la fragmentación en la prestación. Es necesario redefinir el concepto de servicio oncológico bajo el enfoque de atención integral y la importancia de habilitar unidades funcionales, centros integrales de tratamiento y otras formas de atención**.**

## Introduction

About 14 million new cases of cancer ^1^ were recorded in 2012, and the number of cases with cancer incidence is expected to increase by 70% over the next 20 years, facts that have made this disease one of the main causes of morbidity and the second cause of death in the world ^2^, accounting for at least one in every six deaths. In 2015, cancer caused an estimated 8.8 million deaths; close to 70% of deaths have been recorded in low and mid-income countries, where less than 30% of countries provide treatment to patients with an oncological pathology ^3^.

Comprehensive care for cancer patients requires bringing together the various oncological specialties - medical, surgical, radio therapeutic - and it also requires the synergy of a variety of diagnostic services (pathology, clinical laboratory, imaging, nuclear medicine, among others) as well as that of clinical and social support services (nutrition, mental health, social work, pain control, among others) that are complementary ^4^. According to the recommendations made by high-income countries, which have a high number of cancer patients, the oncological surgery, radiotherapy and chemotherapy services should be concentrated in comprehensive treatment centers, which can guarantee a high volume of patients with the same pathology, thus allowing to justify investment in complex treatment technologies, improve medical expertise, and improve clinical outcomes. On the other hand, diagnostic and patient support services must be decentralized ^5^. 

Colombia is a mid-income country, with a cancer incidence rate of 151.5 per 100,000 men and 145.6 per 100,000 women ^6^, with a strong supply of oncological services in the private sector and fragmentation among the services involved in cancer treatment ^4^. That is how, since cancer is a growing public health problem in Colombia, the country placed the Ten-Year Plan for Cancer Control 2012-2021 as part of its public policies, where several goals were defined, including: the need to update the eligibility standards and the oncological services verification modes, as well as the need to organize the service network for comprehensive care of cancer in Colombia ^7^. In meeting this goal, the procedures and conditions for eligibility of health services, including oncology, were regulated in 2014, strengthening the requirements for compliance with quality standards ^8^. 

In Colombia, the Ministry of Health and Social Protection has the power to verify compliance with standards, and technical-scientific conditions for opening and operating a new oncology service, identifying this as a “qualified” service in The Special Registry of Health Service Providers-REPS ^9^. The REPS is the official source of information on the registered offer of health service providers that are authorized to provide health services in each territorial demarcation, which, for the Colombian case, are called departments, according to the political-administrative division of the state. The country identifies two types of health service providers: professionals who provide a single service independently in their private offices and health services provider institutions that offer several health services. The latter group includes hospitals, clinics or similar establishments.

Based on this record, between 2004 and 2012 an increase in private oncological services and an expansion of non-integral services became evident, a fact that clearly showed fragmentation of care ^4^. As of 2012 and after the Ministry of Health and Social Protection regulated the price of oncological medicines, there was a gradual decrease in the number of services provided ^9^, a scenario that possibly slowed down the supply of new oncology services. This was joined by a new national regulation defining the procedures and conditions for registration and authorization of health services, as well as the declaration of mandatory requirement for institutions to obtain a verification by the Ministry of Health ^8^ prior to opening their oncological services, even though the mandatory nature of this verification does not imply that the Ministry regulates the oncological service offering.

In order to avoid care fragmentation, aiming at promoting comprehensive care under the model of units or comprehensive treatment centers has been one of the ways in which the national government has managed to organize the offer. New ways of articulation between oncological services were defined by 2016: Functional Units for Adult Cancer Care-UFCA and Childhood Cancer Care Units-UACAI ^10^, and the creation of Health Service Suppliers’ Comprehensive Networks RIPSS, which the Oncology Services Delivery Network is part of ^11^. Within this context, the objective of this article is to characterize the current oncological services supply status in Colombia and its distribution by departments for the year 2017.

## Materials and Methods

A descriptive analysis of the distribution of oncology services that met the requirements to provide health services for cancer care in the adult and infant population in Colombia, which required prior verification by the Ministry of Health and Social Protection for its operation was conducted, according to information available in the REPS.

The set of oncological services included in the analysis is made up of: outpatient services specialized in the medical and surgical areas, surgical services, and diagnostic support and therapeutic complementation services covering radiotherapy, chemotherapy and nuclear medicine (Table 1) ^8^. The information was consulted with the report prepared by each department up to June 2017.

**Table 1:** Oncology services subject to prior verification by the Ministry of Health and Social Protection.

Group of services	Service code	Service name
Surgical services group	210	Surgical oncology
	227	Pediatric surgical oncology
	232	Breast surgical oncology and soft tissue tumors surgery *
	237	Plastic surgery for the oncological patient
	217	Other surgeries*
Specialized Medical Consultation group	309	Pain and palliative care*
	336	Clinical oncology
	346	Oncology rehabilitation
	364	Breast and soft tissue tumor surgery*
	370	Plastic surgery for the oncological patient
	373	Surgical oncology
	374	Pediatric surgical oncology
	375	Dermatological oncology
	379	Gynecology oncology
	381	Oncology and clinical hematology
	390	Ophthalmic oncology
	391	Pediatric hematology and oncology
	393	Orthopedic oncology
	395	Urologic oncology
	408	Radiotherapy
	383	Nuclear medicine*
	394	Oncologic pathology
	406	Hematological oncology
	356	Other consultations*
Diagnostic and therapeutic support Group	709	Chemotherapy
	711	Radiotherapy
	715	Nuclear Medicine (PET / Iodine therapy)*

* The REPS application has options for the provider to state whether the activities of these services are aimed or not to cancer patients. Source: Resolution 2003 of 2014.

We conducted the search by using the “guest” user access profile. We enter the “Current REPS” module, in the services item. Two search criteria were used: name and code of the services of interest, according to the service structure set forth in Resolution 2003 of 2014 (Table 1).

We carried out an information selection process, which included all the oncological services registered in the REPS, except those services that stated a “non-oncological” focus when registered, that is, that the activities to be developed by them were not aimed at dealing with cancer patients. The services corresponding to "other" name codes were incorporated into the analysis only in those cases in which the term oncological specialty was specified in the service name. The variables defined in the analysis were those related to geographical distribution, service group, legal nature, type of provider, level of care, territorial character, complexity and locations. Variables related to the provision mode were excluded based on incomplete information.

The case-by-provider ratio was calculated from cancer incidence data estimated for Colombia, information published by the National Institute of Cancer - INC ^6^, on the number of qualified oncological IPS. Calculations for 27 departments, the Capital District and the Amazonas group (Amazonas, Guainía, Guaviare, Vaupés and Vichada) were made. In addition, the correlation coefficient between new cancer cases per year and the number of oncological IPS was calculated.

## Results

We identified 1,780 qualified health services in the national territory related to specialties for cancer patient care, as well as close to 63,000 new patients per year, according to estimates of the INC regarding cancer incidences in the country.

### Offer of health services by geographic location

**Figure 1:**
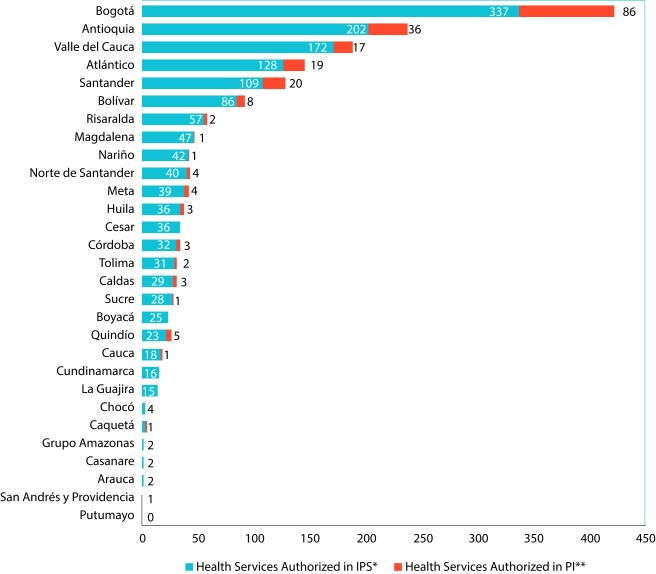
Qualified Oncology Services by provider class and Department. Cut-off date: June 30, 2017. **Source:** Database of the Special Register of Health Service Providers REPS. Ministry of Health and Social Protection. . * IPS: Institution providing health services, ** PI: Independent professional.

We found records of oncological services in 28 departments, with at least one provider with an authorized service in each territorial demarcation. The departments of Putumayo and the Amazonas group, with the exception of Vaupés, did not record any oncological services. Nearly 70% of the country's offer was concentrated in the Capital District, Bogotá D.C**.** (23.8%) and in the departments of Antioquia (13.4%), Valle del Cauca (10.6%), Atlántico (8.3%), Santander (7.2%) and Bolívar (5.3%) (Fig. 1).

With the exception of Chocó and Santander, capital cities in most departments offered over 85% of oncological services available in each territory. Bogotá, D.C., Medellin and Cali stood out as the main urban centers with a high number of health services for oncological diseases care. In the case of Chocó, its capital Quibdó provided 75% of the oncological offer; as for Santander, Bucaramanga offers about half of all oncology services (48%) and the remaining offer is provided, in descending order, by the municipalities of Piedecuesta, Floridablanca (Metropolitan Area) and Barrancabermeja.

### Offer of oncological services by type of provider, legal nature and level of care

Out of 1,780 qualified services provided in the country, 87.8% (1,563 services) was offered by Healthcare Providing Institutions - IPSs, and the remaining 12.2% (217 services) was provided by independent professionals - PI (Table 2).

**Table 2:** Ratio between new cases of cancer and IPSs with oncology services in departments of Colombia. Cut-off date: June 30, 2017.

Department	Estimated incidence	Oncology IPS	Cases/ *IPS ratio
Casanare	309	1	309
Antioquia	9,781	34	288
Arauca	253	1	253
Boyacá	1,813	7	259
Cauca	1,521	6	254
Tolima	2,308	100	231
Valle del Cauca	7,639	353	218
Córdoba	1,356	8	170
Caldas	1,860	11	169
Norte de Santander	1,815	11	165
Amazonas Group	164	14	164
Risaralda	1,723	11	157
Bogotá	11,068	72	154
Nariño	1,810	121	151
Caquetá	447	3	149
Quindío	1,172	8	147
Huila	1,451	10	145
Meta	1,206	9	134
Chocó	279	2	140
Sucre	737	67	123
Santander	2,961	25	118
Cesar	990	9	110
Cundinamarca	3,157	3	105
Magdalena	1,249	15	83
Bolívar	2,019	25	81
Atlántico	3,010	37	81
San Andrés y Providencia	78	1	78
La Guajira	440	8	55
Putumayo	202	0	0
Colombia	62,818	38,118	165

Source: REPS database.* IPS: Institution providing health care services.

The IPSs group makes up 381 institutions and 443 care-providing centers; this means that some of those IPSs enabled oncology services in more than one location. According to their legal nature, 91.1% of these institutions were private, 7.9% were public companies and only 1.0% were mixed entities. Regarding service provision, 362 IPSs (95%) stated to be medium and high complexity and 19 low complexity IPSs. In addition, 21 IPSs recorded that they provide services in the third level of care, only 9 IPS in the second level of care, and the rest of IPSs did not differentiate their level of service provision.

As for the 347 existing private IPSs nationwide, they managed 1,374 of the 1,563 services authorized at IPSs, that is, 87.9% of the oncology services supply in Colombia is provided by private sector IPSs.

Public IPSs, which manage 180 services, correspond mostly to institutions of departmental coverage (70%), and a smaller number of entities have coverage at the national (10%), district (10%) and municipal (10%) levels. Likewise, the 30 public IPSs are entities that depend directly on the state or the departments, except for two institutions belonging to the special regime of military health and the national police.

Regarding independent professionals (195), it was found that several of them enabled more than one service or the same service in different locations. The average of independent professionals by department is seven, it is important to note that in Arauca, Boyacá, Casanare, Cesar, Chocó, Cundinamarca, La Guajira and San Andrés and Providencia departments no oncology services enabled under this type of providers (independent professionals) were found.

In general, Colombia had 576 health service providers to serve the 62,818 new cases of cancer per year estimated in the country, with an average of 2,166 cases of cancer per territory and 20 providers on average to meet this demand.

From the comprehensive care at the IPSs standpoint, there are an average of 13 IPSs per territory, with an average of 4.1 oncology services enabled per institution. This was the offer available for 165 new cases per year by IPS, with a range of variation between 55 cases in institutions located in La Guajira and 1,052 cases to be addressed per IPSs located in Cundinamarca (Table 2).

**Figure 2:**
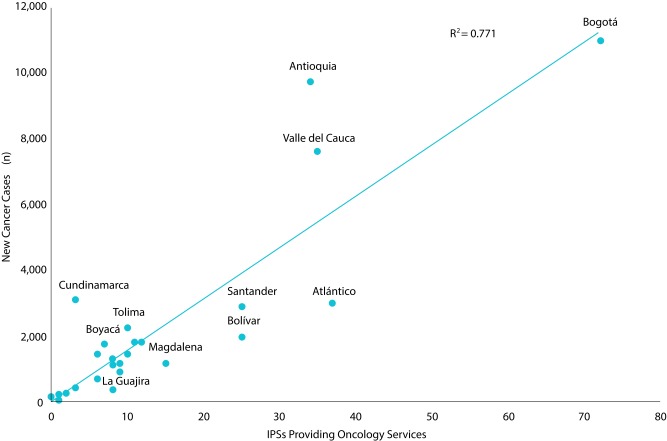
Ratio of estimated incidence by departments and IPSs providing oncology services in Colombia.

The number of new cancer cases estimated by department showed a positive ratio with the number of oncology IPSs (r= 0.87). Some departments such as Antioquia, Valle del Cauca and Cundinamarca have new cases of cancer by IPS above the national average (165). The opposite is shown for the Departments of the Caribbean region and Santander (Fig. 2).

### Offer according to health service groups

When differentiating services according to the group classification structure, it was found that more than half of all oncology services were outpatient services (66.7%), and there was a lower percentage of participation in the offer for services related to the diagnostic support and therapeutic support group (17.4%), and the surgical one (15.9%) (Table 3).

**Table 3:** Group of qualified (authorized) oncology services. Cut-off-date: June 30, 2017.

Service group	Service name	Number of services enabled per IPS *	Number of services enabled per PI**
Surgical services group	Surgical oncology	105	0
	Pediatric surgical oncology	20	0
	Breast surgical oncology and surgery of soft tissue tumors *	122	0
	Plastic surgery for the oncological patient	31	0
	Other surgeries - Surgical oncology	3	0
	Other surgeries- Gynecology oncology and mastology	1	0
	Other surgeries- Orthopedic oncology	1	0
Specialized medical consultation group	Pain and palliative care	132	15
	Clinical oncology	182	37
	Rehabilitation oncology	12	0
	Breast and soft tissue tumor surgery	87	30
	Plastic surgery for the oncological patient	24	8
	Surgical oncology	83	21
	Pediatric surgical oncology	10	0
	Dermatological oncology	15	5
	Gynecology oncology	113	44
	Oncology and clinical hematology	0	0
	Ophthalmic oncology	13	4
	Pediatric hematology and oncology	68	2
	Orthopedic oncology	38	6
	Urology oncology	33	10
	Radiotherapy	46	14
	Nuclear medicine	22	0
	Oncologic pathology	0	0
	Hematological oncology	87	8
	Other consultations- Oncology	9	9
Diagnostic support and therapeutic complementation group	Chemotherapy	180	0
	Radiotherapy	51	2
	Nuclear Medicine (PET / Iodine therapy)	75	2

Source: REPS database.

* IPS: Institution providing health services,

** PI: Independent professional.

The outpatient group showed 1,187 services for 16 oncology specialties, among which clinical oncology, oncological gynecology, pain and palliative care, and breast surgery and surgery of soft tissue tumors stands out because of their higher availability; these consultations account for over 50% of the total offer of this group of services. 82.1% of outpatient services were located in IPSs, and 17.9% of outpatient consultations were provided by independent professionals.

The diagnostic support and therapeutic support group are made up of services providing the traditional treatment modalities for cancer: chemotherapy and radiotherapy and nuclear medicine. In absolute figures, the services attached to this group were 310, discriminated as follows: chemotherapy (180), nuclear medicine (77) and radiotherapy (53). Participation of independent professionals in the offer of this group was 1.3%, which means that 98.7% of qualified diagnostic and therapeutic services in oncology are provided by IPSs.

In addition, an offering consisting of six chemotherapy services was found in IPSs that did not have any outpatient services in clinical oncology or hematology and pediatric oncology.

The surgical group showed a total of 283 services, where breast and soft tissue surgery account for 43.1% of the total offered by the group. Therefore, the availability of other surgical services, in descending order, was: general oncological surgery (37.1%), oncological plastic surgery (11.0%), pediatric oncological surgery (7.1%), oncological gynecological surgery and mastology (0.4%), oncological orthopedics surgery (0.4%), and other oncological surgeries undifferentiated by specialty in the record (1.1%).

With regard to comprehensiveness of health services involved in conventional forms of cancer treatment (chemotherapy, radiotherapy, surgery), we found 257 providers offering these services, of which 65.3% offered one of these three services, therefore 88 providers only offered surgical oncology services, 77 providers only offered services to administer chemotherapy and 9 providers had radiotherapy services exclusively for diagnostic support and therapeutic complementation. Also, a percentage of 24.9% of this group of providers offered two services for treatment, which means that 49 providers had chemotherapy and oncological surgery services, and 15 providers offered chemotherapy and radiotherapy services. In summary, 25 providers nationwide had the three services available: chemotherapy, radiotherapy and surgery.

## Discussion

This descriptive study presents the characterization of oncological services in Colombia to provide care to cancer patients, based on information available up to June 2017. A high concentration of oncological services was found in the capital cities, out of which more than half are related to outpatient services (67%) and 88% of them are private. Only 25 providers nationwide had the three services available: chemotherapy, radiotherapy and surgery. Although the development of care models under the figure of comprehensive units of care and networks has been deemed as essential, it is worth noting that no records were found so far regarding functional clinical units for adult cancer-UFCA, or units for Comprehensive Care of Childhood Cancer UACAI, and also no Comprehensive Networks for the Provision of Health Services were found; according to this, thinking about a harmonization of the service network in the different levels of complexity that guarantees a quality and opportunity in the diagnosis of the oncological disease is still somehow complicated; this clearly shows the need the country has to classify and redefine what is currently defined as an oncological service ^12^. 

The study clearly shows that there are large differences in the number of new cases of cancer in different regions, and that the largest numbers of cases are in the departments of Antioquia, Atlántico, Valle del Cauca, Cundinamarca, and Bogotá. Incidence estimates show that the demand for services varies according to the geographical characteristics, and this reason would usually consider that the offer of oncological services should be in accordance with local demand. However, as it has already been shown in the world, a case-by-case ratio of 165 cases per IPS per year, as found in this study is very low to guarantee successful health outcomes, considering that comprehensive treatment centers such as the National Cancer Institute INC, handles about 7,000 new cases per year; nevertheless, this fact is special because this is a reference institution that offers care to patients from all over the country and does not segment its offer to regions. ^4^. Likewise, countries such as the United Kingdom, which has an incidence of 273 (APR per 100,000 person-years), states that there must be a comprehensive treatment center for every 2.5 to 3.6 million people ^13^, a concept that if brought to the Colombian context, would mean having more or less 15 comprehensive centers offering a multitude of oncological services, which is far from the current situation in the country.

In the last decade, the offer of oncological health services in Colombia showed a trend oriented to enabling services of the group of consultation of oncological medical specialties, with second and third places for services of diagnosis and therapeutic and surgical complementation support; as a result of this dynamics, over half of oncology services are outpatient services, more than 65% of providers offering cancer treatment services tend to offer only one type of service, and only 6.5% of IPSs have comprehensive chemotherapy, radiotherapy and oncological surgery services. This lack of balance in the ratio of service groups and the low average of services provided by the IPSs suggests the existence of health centers that do not integrate the basic oncological therapeutic modalities, which hinders institutional coordination and sets up barriers to access as well as quality in the care provided ^14^. 

Even though this article is not an analysis of sufficiency of oncological services by specialty, since it includes variables referring to the productive capacity of the services (infrastructure, human talent, production, times and movements), the analysis certainly showed that the gynecology and breast surgery services as well as those for soft tissue tumors were the specialties with the highest offer in consultation, data concomitant with the main types of cancer in women (breast and cervix). In the case of oncological gynecology, its high offer does reflect the priority granted to providing care for the pathologies treated by specialty, therefore, with the current offer, each authorized oncology gynecology consultation service handles an average of 30 new cases of cervix cancer per year out of the approximately 5,000 cases diagnosed; nevertheless, breast consultation is not necessarily enabled for cancer care, so the figures do not tacitly correspond to the offer for treatment of an oncological pathology. In addition, if we consider that of the 29,734 new cases of cancer per year that occur in men ^6^, prostate cancer is the most frequent with around 9,000 cases, but the offer of outpatient care for oncological urology was low, since a consultation service for this specialty examines an average of 210 new cases of prostate cancer per year, a number that is far away from the figure observed for women's care.

Additionally, the record showed that, put together, the offer for consultations of the oncological specialties of dermatology, rehabilitation, ophthalmology, nuclear medicine and pediatric surgery, does not account for more than 5% of the total offer of the outpatient services group.

All the services of the surgical group were enabled by IPSs, otherwise this would not be feasible, given the criteria of the interdependence standard for qualification of surgical services according to Resolution 2003 of 2014, a standard that regulates qualification of health services. Some radiotherapy and nuclear medicine services for diagnostic support and therapeutic complementation were enabled by independent professionals; however, given the requirements of the human talent standard of the qualifying standard, this is a non-viable condition, since operation of these services involves several professionals and therefore cannot be offered by a single independent professional. This fact reflects discrepancies in the information system of the registry of providers in the REPS.

In advanced oncological disease, pain relief and palliative care is the only realistic treatment option that allows for improving quality of life ^14^. Palliative care along with other disciplines such as rehabilitation, nutrition and mental health are fundamental areas of care in all phases of the disease and make up the oncological support services, as proposed by the *Cancer patient care Model* ^4^; thus, pain and palliative care consultation was the third most offered and, among all consultations, oncological rehabilitation was the third least offered, these figures show the need to strengthen these support services. Although this deduction is subject exclusively to the classification of services in the registry of service providers, it does not exclude the fact that rehabilitation is incorporated during the service provided, and that services are recorded as “general” rehabilitation. Similarly, palliative care and pain counseling are not exclusive in cancer treatment, so the offer for this specialty in oncology may be overrated.

### Strengths and Limitations

The availability of an information system that allows characterizing offer of health services in an up-to-date manner is a tool of great value for all actors in the health system, including providers, patients and decision-makers. This advantage makes it possible to differentiate the oncological approach of some specialties in such a way that the characterization of these services in particular turns out to be as close as possible to the real offer available for cancer care.

However, the quality of the information contained in the registry of providers has some irregularities. Data asymmetry is a limitation that can be explained by the singularity in the registration process of providers in the registry. One of the causes most often observed is the possibility that providers have to register services under codes framed in the so-called “other”, which is why many specialties that provide outpatient and surgical services enable their services in an undifferentiated way and generate the relative divergences observed amongst the oncological subspecialties offer. As an addition to this limitation, radiotherapy and nuclear medicine services, both for outpatient consultation and diagnostic support and therapeutic complementation, were registered years ago under the same service code, which did not allow them to be differentiated according to their clinical purpose. Therefore, it is possible these services have not yet been updated to the new registration codes and are wrongly classified. So, the data presented should be dealt with exercising caution.

Finally, the data provided by the REPS showed some per se flaws in the process of registration and verification of services, and this limits the analysis of variables that can provide relevant data. Therefore, it is required to update the information related to service providers, so as to have a closer and more realistic view of the real situation of the provision of oncological services in Colombia.

## Conclusion

Colombia is a country with a wide and extensive range of oncological services throughout the national territory, with service nuclei identified mainly in capital cities. The core of oncological services offer is concentrated in private providers with a minimum participation of public entities belonging to the Colombian state. In general, the ratio between groups of services is asymmetric, the majority of them being oncological outpatient services with few providers that offer together the basic services for oncology treatment, which reflects how fragmented provision is, a fact that definitely does not benefit the patient. It is therefore necessary to redefine the concept of oncology service under the comprehensive care approach and the importance of authorizing or enabling units, comprehensive treatment centers and other forms of care that guarantee quality care with accessibility, comprehensiveness and continuity. The capacity of oncological services for the current and future needs of the country is not yet exactly known.
