# Supplementary material for: Oncology services supply in Colombia
Source: Colomb Med (Cali). 2018 Mar 30;49(1):89–96. doi: 10.25100/cm.v49i1.3620 (PMC6018814; doi:10.25100/cm.v49i1.3620)
Supplement: Related texts

# Research article: Servicios oncológicos en Colombia

## Introducción

En 2012 se registraron alrededor de 14 millones de casos nuevos de cáncer ^1^ y se prevé que en los próximos 20 años, el número de casos incidentes con cáncer aumente en un 70%, hecho que ha convertido esta enfermedad en una de las principales causas de morbilidad y la segunda causa de muerte en el mundo ^2^, atribuyéndole por lo menos una de cada seis defunciones. En el 2015 ocasionó un estimado 8.8 millones de fallecimientos, cerca del 70% de las muertes se registraron en países de ingresos medianos y bajos, donde menos del 30% de los países ofrecen tratamiento a los pacientes con patología oncológica ^3^**.**

La atención integral del paciente con cáncer requiere de la articulación de las diferentes especialidades oncológicas -médicas, quirúrgicas, radioterápicas - así como de la sinergia de una diversidad de servicios diagnósticos (patología, laboratorio clínico, imagenología, medicina nuclear entre otros) y de soporte clínico y social (nutrición, salud mental, trabajo social, control del dolor entre otros) que son complementarios ^4^. De acuerdo con las recomendaciones de los países de altos ingresos, que tienen una alta carga de pacientes con cáncer, los servicios de cirugía oncológica, radioterapia, quimioterapia deben estar concentrados en centros integrales de tratamiento. Estos centros garantizan un alto volumen de pacientes con una misma patología y permite justificar la inversión en complejas tecnologías de tratamiento, mejorar la experticia médica así como mejorar los desenlaces clínicos. Por el contrario los servicios de diagnóstico y soporte al paciente deben estar descentralizados ^5^. 

Colombia es un país de ingresos medios con una tasa de incidencia de 151.5 por 100,000 hombres y 145.6 por 100,000 mujeres ^6^, con una marcada oferta de servicios oncológicos en el sector privado y fragmentación entre los servicios que intervienen en el tratamiento del cáncer ^4^**.** Es así como siendo el cáncer un problema de salud pública creciente para Colombia, el país posicionó en la agenda pública el Plan decenal para el control del cáncer 2012-2021, donde se definieron varias metas dentro de la cual se encuentra: la necesidad de actualizar los estándares de habilitación y los modos de verificación de los servicios oncológicos así como la de organizar la red de servicios para la atención integral del cáncer en Colombia ^7^. En cumplimiento de esta meta, en el año 2014 se reglamentaron los procedimientos y condiciones de habilitación de servicios de salud, incluidos los oncológicos, en los que se fortalecen los requisitos de cumplimiento de los estándares de calidad ^8^. 

En Colombia, el Ministerio de Salud y de la Protección Social es quien tiene la potestad de verificar el cumplimiento de los estándares, condiciones técnico- científicas, para la apertura y funcionamiento de nuevos servicios de oncología, identificándolo como servicio ‘’habilitado’’ en El Registro Especial de Prestadores de Servicios de Salud-REPS ^9^. El REPS, es la fuente oficial de información sobre la oferta registrada de los prestadores de servicios de salud que se encuentran autorizada para prestar servicios de salud en cada demarcación territorial, para el caso Colombiano denominado departamento, según la división político-administrativa del estado. El país identifica dos tipos de prestadores de servicios de salud, los profesionales que prestan un único servicio de forma independiente en sus consultorios particulares y las instituciones prestadoras de servicios de salud que prestan varios servicios de salud. Estas últimas agrupan los hospitales, clínicas u otro establecimiento similar.

Con base en este registro, entre los años 2004-2012 se hizo evidente un aumento de servicios oncológicos privados y una expansión de servicios no integrales, hecho que hacía clara la fragmentación de la atención ^4^. A partir del año 2012 y después que el Ministerio de Salud y Protección social regulara el precio a los medicamentos oncológicos se registró un descenso gradual en el número de servicios habilitados ^9^, panorama que posiblemente desaceleró la oferta de nuevos servicios oncológicos. A lo anterior se sumó una nueva reglamentación nacional que definió los procedimientos y condiciones de inscripción y de habilitación de los servicios de salud así como la declaración de la obligatoriedad que tienen las instituciones de obtener una verificación previa a la apertura de servicios oncológicos por parte del Ministerio de salud ^8^, no obstante esta obligación de verificación no implica que el Ministerio regule la oferta de servicios oncológicos. 

Una de las formas en que el gobierno nacional ha gestionado la organización de la oferta, para evitar la fragmentación de la atención, es la búsqueda de la promoción de la atención integral bajo el modelo de unidades o centros integrales de tratamiento. Para el año 2016 se definieron nuevas formas de articulación entre servicios oncológicos: la definición de las Unidades Funcionales para la Atención de Cáncer del Adulto-UFCA y las Unidades de Atención de Cáncer infantil-UACAI ^10^, y la conformación de las Redes Integrales de Prestadores de Servicios de Salud-RIPSS, de la cual hace parte la Red de Prestación de Servicios Oncológicos ^11^. Dentro de este contexto, el objetivo de este artículo fue caracterizar la situación actual de la oferta de servicios oncológicos en Colombia y su distribución por departamentos para el año 2017.

## Materiales y Métodos

Se realizó un análisis descriptivo de la distribución de la oferta de servicios oncológicos que cumplieron con los requisitos para prestar servicios de salud para la atención de cáncer en población adulta e infantil en Colombia, y requirieron verificación previa por parte del Ministerio de Salud y Protección Social para su funcionamiento, de acuerdo a la información disponible en el REPS. 

El conjunto de servicios oncológicos incluido en el análisis comprende: servicios de consulta externa especializadas en las áreas médicas y quirúrgicas, servicios quirúrgicos, y los de apoyo diagnóstico y complementación terapéutica que abarcan radioterapia, quimioterapia y medicina nuclear (Tabla 1) ^8^. La consulta de la información se realizó con el reporte elaborado por cada departamento al mes de Junio de 2017.

**Tabla 1:** Servicios oncológicos objeto de verificación previa por parte del Ministerio de Salud y Protección Social.

Grupo de servicios	Código del servicio	Nombre del servicio
Grupo servicios quirúrgicos	210	Cirugía oncológica
	227	Cirugía oncológica pediátrica
	232	Cirugía de mama y tumores de tejidos blandos *
	237	Cirugía plástica oncológica
	217	Otras cirugías*
Grupo Consulta médica especializada	309	Dolor y cuidados paliativos*
	336	Oncología clínica
	346	Rehabilitación oncológica
	364	Cirugía de mama y tumores de tejidos blandos*
	370	Cirugía plástica oncológica
	373	Cirugía oncológica
	374	Cirugía oncológica pediátrica
	375	Dermatología oncológica
	379	Ginecología oncológica
	381	Oncología y hematología clínica
	390	Oftalmología oncológica
	391	Oncología y hematología pediátrica
	393	Ortopedia oncológica
	395	Urología oncológica
	408	Radioterapia
	383	Medicina nuclear*
	394	Patología oncológica
	406	Hematología oncológica
	356	Otras consultas*
Grupo apoyo diagnóstico y complementación terapéutica	709	Quimioterapia
	711	Radioterapia
	715	Medicina Nuclear (PET / Yodoterapia)*

* El aplicativo del REPS tiene dispuestas las opciones para que el prestador declare si las actividades de éstos servicios se dirigen o no al manejo del paciente oncológico. Fuente: Resolución 2003 de 2014.

La búsqueda se efectuó con perfil de acceso de usuario “invitado”. Se ingresó al módulo “REPS vigente”, en el ítem de servicios. Se utilizaron dos criterios de búsqueda: nombre y código de los servicios de interés, de acuerdo con la estructura de servicios estipulada en la Resolución 2003 de 2014 (Tabla 1). 

Se realizó un proceso de selección de información, en el que se incluyeron todos los servicios oncológicos registrados en el REPS, con excepción de los servicios que declararon en su inscripción su enfoque “no oncológico”, es decir, que las actividades a desarrollar por estos no están dirigidas al manejo del paciente con cáncer. Los servicios correspondientes a códigos de denominación “otras” fueron incorporados al análisis sólo en los casos en que se detalló en el nombre del servicio o el apelativo de especialidad oncológica. Las variables definidas en el análisis fueron las relacionadas con distribución geográfica, grupo de servicio, naturaleza jurídica, clase de prestador, nivel de atención, carácter territorial, complejidad y sedes. Se excluyeron variables referentes a la modalidad de prestación por presentar información incompleta.

La razón de casos por prestador se calculó a partir de los datos de incidencia de cáncer estimada para Colombia, información publicada por el Instituto Nacional de Cancerología - INC ^6^, sobre el número de IPS oncológicas habilitadas. Los cálculos se generaron para 27 departamentos, el Distrito capital y el grupo Amazonas (Amazonas, Guainía, Guaviare, Vaupés y Vichada). Además se calculó el coeficiente de correlación entre los casos de cáncer nuevos al año y el número de IPS oncológicas.

## Resultados

Se identificaron 1,780 servicios de salud habilitados en el territorio nacional relacionados con especialidades para la atención de pacientes oncológicos y cerca de 63,000 pacientes nuevos anuales, según estimaciones de la incidencia del cáncer para el país en el INC. 

### Oferta de servicios de salud por ubicación geográfica

La oferta de servicios oncológicos se registró en 28 departamentos con la existencia de por lo menos un prestador con un servicio habilitado en cada demarcación territorial. Los departamentos de Putumayo y del grupo Amazonas, con excepción de Vaupés, no registraron servicios oncológicos. Cerca del 70% de la oferta del país se concentró en el Distrito Capital, Bogotá (23.8%) y los departamentos de Antioquia (13.4%), Valle del Cauca (10.6%), Atlántico (8.3%), Santander (7.2%) y Bolívar (5.3%) (Fig. 1). 

**Figura 1:** Servicios Oncológicos habilitados por clase de prestador y Departamento. Fecha de corte: Junio 30 de 2017. **Fuente:** Base de datos del Registro Especial de Prestadores de Servicios de salud REPS. Ministerio de Salud y Protección Social. .* IPS: Institución prestadora de servicios de salud, **PI: Profesional independiente

Las ciudades capitales en la mayoría de los departamentos con excepción de Chocó y Santander, ofertaron más del 85% de servicios oncológicos disponibles en cada territorio. Se destacaron Bogotá, D.C., Medellín y Cali como los principales núcleos urbanos que contaron con un alto número de servicios de salud para la atención de la enfermedad oncológica. Para el caso de Chocó, su capital Quibdó proveía el 75% de la oferta oncológica; para Santander, Bucaramanga ofrece cerca de la mitad de los servicios oncológicos (48%) y la oferta restante es asumida, en orden descendente, por los municipios de Piedecuesta, Floridablanca (Área metropolitana) y Barrancabermeja. 

### Oferta de servicios oncológicos por clase de prestador, naturaleza jurídica y nivel de atención

De los 1,780 servicios habilitados en el país, el 87.8% (1,563 servicios), fue ofertado por Instituciones Prestadoras de Salud - IPS, y el 12.2% excedente (217 servicios) fue provisto por profesionales independientes (Tabla 2). 

**Tabla 2:** Relación entre casos nuevos de cáncer e IPS con servicios oncológicos en departamentos de Colombia. Fecha de corte: Junio 30 de 2017.

Departamento	Incidencia estimada	IPS oncología	Razón casos/ *IPS
Casanare	309	1	309
Antioquia	9,781	34	288
Arauca	253	1	253
Boyacá	1,813	7	259
Cauca	1,521	6	254
Tolima	2,308	100	231
Valle del Cauca	7,639	353	218
Córdoba	1,356	8	170
Caldas	1,860	11	169
Norte de Santander	1,815	11	165
Grupo Amazonas	164	14	164
Risaralda	1,723	11	157
Bogotá	11,068	72	154
Nariño	1,810	121	151
Caquetá	447	3	149
Quindío	1,172	8	147
Huila	1,451	10	145
Meta	1,206	9	134
Chocó	279	2	140
Sucre	737	67	123
Santander	2,961	25	118
Cesar	990	9	110
Cundinamarca	3,157	3	105
Magdalena	1,249	15	83
Bolívar	2,019	25	81
Atlántico	3,010	37	81
San Andrés y Providencia	78	1	78
La Guajira	440	8	55
Putumayo	202	0	0
Colombia	62,818	38,118	165

El conjunto de IPS estuvo conformado por 381 instituciones y 443 sedes, lo cual indica que algunas IPS habilitaron servicios oncológicos en más de una sede. Según su naturaleza jurídica, el 91.1% de estas instituciones eran de carácter privado, el 7.9% empresas públicas y sólo el 1.0% entidades mixtas. En relación con la prestación de los servicios, 362 IPS (95%) se declararon de mediana y alta complejidad y 19 IPS de baja complejidad. Además, 21 IPS registraron la prestación de servicios en el tercer nivel de atención, únicamente 9 IPS en el segundo nivel de atención, y el resto de IPS no diferenciaron su nivel de prestación. 

En cuanto a las 347 IPS privadas existentes en el orden nacional administraban 1,374 de los 1,563 servicios habilitados en IPS, es decir, el 87.9% de la oferta de servicios oncológicos en Colombia está en el sector privado. 

Las IPS de carácter público con la administración de 180 servicios, corresponden en su mayoría a instituciones de dominio departamental (70%), y un número menor de entidades tienen competencias a nivel nacional (10%), distrital (10%) y municipal (10%). Así mismo, las 30 IPS públicas son entidades que dependen directamente del estado o de los departamentos, salvo dos instituciones pertenecientes al régimen especial de sanidad militar y de la policía nacional. 

En relación con los profesionales independientes (195), se encontró qué varios de ellos habilitaron más de un servicio o el mismo servicio en distinta sede. El promedio de profesionales independientes por departamento es de siete, con la observación de que en los departamentos de Arauca, Boyacá, Casanare, Cesar, Chocó, Cundinamarca, La Guajira y San Andrés y Providencia no hubo servicios oncológicos habilitados bajo la figura de estos prestadores. 

En general, para la atención de los 62,818 casos nuevos de cáncer por año estimados para el país, Colombia contaba con 576 prestadores de servicios de salud, con un promedio por territorio de 2,166 casos de cáncer y 20 prestadores promedio para atender esta demanda.

Desde la perspectiva de la atención integral en las IPS, se evidenció una media de 13 IPS por territorio, con un promedio de 4.1 servicios oncológicos habilitados por institución. Esta oferta era la disponible para la atención de 165 casos nuevos al año por IPS, con un rango de variación entre 55 casos en instituciones ubicadas en La Guajira y 1,052 casos para atender por IPS localizadas en Cundinamarca. (Tabla 2). 

El número de casos de nuevos de cáncer estimados por departamento mostró una relación positiva con el número de IPS oncológicas (r = 0.87). Algunos departamentos como Antioquia, Valle del Cauca y Cundinamarca tienen un número de casos nuevos de cáncer por IPS por encima del promedio nacional (165). Caso contrario se presenta en los Departamentos de la región caribe y Santander (Fig. 2).

**Figura 2:** Relación entre la incidencia estimada por departamentos e IPS oncológicas en Colombia. Fuente: Base de datos del REPS.* IPS: Institución prestadora de servicios de salud.

### Oferta según los grupos de servicios de salud 

Al hacer la distinción de los servicios de acuerdo con la estructura de clasificación por grupos, se encontró que más de la mitad de los servicios oncológicos eran de consulta externa (66,7%), y un porcentaje menor de participación en la oferta para los servicios del grupo de apoyo diagnóstico y complementación terapéutica (17.4%), y quirúrgicos (15.9%) (Tabla 3). 

**Tabla 3:** Grupo de servicios oncológicos habilitados. Fecha de corte: Junio 30 de 2017.

Grupo de servicios	Nombre del servicio	Número de servicios habilitados por IPS*	Número de servicios habilitados por PI**
Grupo servicios quirúrgicos	Cirugía oncológica	105	0
	Cirugía oncológica pediátrica	20	0
	Cirugía de mama y tumores de tejidos blandos	122	0
	Cirugía plástica oncológica	31	0
	Otras cirugías- Si oncológicas	3	0
	Otras cirugías- Ginecología oncológica y mastología	1	0
	Otras cirugías- Ortopedia oncológica	1	0
Grupo consulta médica especializada	Dolor y cuidados paliativos	132	15
	Oncología clínica	182	37
	Rehabilitación oncológica	12	0
	Cirugía de mama y tumores de tejidos blandos	87	30
	Cirugía plástica oncológica	24	8
	Cirugía oncológica	83	21
	Cirugía oncológica pediátrica	10	0
	Dermatología oncológica	15	5
	Ginecología oncológica	113	44
	Oncología y hematología clínica	0	0
	Oftalmología oncológica	13	4
	Oncología y hematología pediátrica	68	2
	Ortopedia oncológica	38	6
	Urología oncológica	33	10
	Radioterapia	46	14
	Medicina nuclear	22	0
	Patología oncológica	0	0
	Hematología oncológica	87	8
	Otras consultas- Si oncológico	9	9
Grupo apoyo diagnóstico y complementación terapéutica	Quimioterapia	180	0
	Radioterapia	51	2
	Medicina Nuclear (PET / Yodoterapia)	75	2

Fuente: Base de datos del REPS.

* IPS: Institución prestadora de servicios de salud

**PI: Profesional independiente

El grupo de consulta externa presento 1,187 servicios para 16 especialidades oncológicas, en las que se destacan por su más alta disponibilidad, oncología clínica, ginecología oncológica, dolor y cuidados paliativos y cirugía de mama y tumores de tejidos blandos ; estas consultas abarcan más del 50% de la oferta total de este grupo de servicios. El 82.1% de los servicios de consulta externa se ubicaron en IPS, y el 17.9% de las consultas se ofertaron por los profesionales independientes. 

El grupo de apoyo diagnóstico y complementación terapéutica está integrado por los servicios con las modalidades terapéuticas de tratamiento tradicional del cáncer: quimioterapia y radioterapia y medicina nuclear. En cifras absolutas, los servicios adjuntos a este grupo fueron 310 discriminados así: quimioterapia (180), medicina nuclear (77) y radioterapia (53). La participación de los profesionales independientes en la oferta de este grupo fue de 1.3%, así que el 98.7% de la capacidad habilitada de servicios diagnósticos y terapéuticos en oncología se encuentro en las IPS. 

Además, se observó la oferta de seis servicios de quimioterapia en IPS que no contaban con servicios de consulta externa en oncología clínica o hematología y oncología pediátrica. 

El grupo quirúrgico se consolidó con 283 servicios, donde cirugía de mama y de tumores blandos correspondió al 43.1% del total ofertado por el grupo. Por consiguiente, la disponibilidad de los otros servicios quirúrgicos, en orden descendente, fueron: cirugía oncológica general (37.1%), cirugía plástica oncológica (11.0%), cirugía oncológica pediátrica (7.1%), cirugía ginecológica oncológica y mastología (0.4%), cirugía de ortopedia oncológica (0.4%) y otras cirugías oncológicas no diferenciadas por especialidad en el registro (1.1%). 

En consideración a la integralidad de servicios de salud involucrados en las formas convencionales de tratamiento del cáncer (quimioterapia, radioterapia, cirugía), se halló oferta de estos servicios en 257 prestadores, de los cuales el 65.3% oferto uno de estos tres servicios, de modo que, 88 prestadores ofertaron únicamente servicios quirúrgicos oncológicos, 77 prestadores tenían habilitados solo servicios para administración de quimioterapia y 9 prestadores disponían de servicios de radioterapia exclusivos para apoyo diagnóstico y complementación terapéutica. Asimismo, un porcentaje de 24.9% de este grupo de prestadores oferto dos servicios para el tratamiento, así, 49 prestadores disponían de servicios de quimioterapia y cirugía oncológica, y 15 prestadores ofertaron en conjunto servicios de quimioterapia y radioterapia. En suma, 25 prestadores a nivel nacional contaron con los tres servicios habilitados: quimioterapia, radioterapia y cirugía.

## Discusión

Este estudio descriptivo presenta la caracterización de los servicios oncológicos en Colombia para la atención de los pacientes con cáncer, con información disponible a junio 2017. Se encontró una alta concentración de servicios oncológicos en las ciudades capitales del país, siendo más de la mitad de ellos servicios de consulta externa (67%) y el 88% de ellos de naturaleza privada. Solamente, 25 prestadores a nivel nacional contaron con los tres servicios habilitados: quimioterapia, radioterapia y cirugía. Aunque se ha considerado imprescindible el desarrollo de modelos de atención bajo la figura de unidades integrales de atención **y** redes, es de observar que hasta el momento, no se encontraron registros de Unidades clínicas funcionales para cáncer adulto-UFCA ni de Unidades para la atención integral del cáncer infantil UACAI y tampoco de Redes Integrales de Prestación de servicios de Salud; siendo así, resulta complejo pensar en la armonización de la red de servicios en los distintos niveles de complejidad que garanticen una calidad y oportunidad en el diagnóstico de la enfermedad oncológica; esto muestra claramente la necesidad que requiere el país de hacer una clasificación y redefinición de lo que se define actualmente como servicio oncológico ^12^. 

Es claro, por el estudio, que existen grandes diferencias en el número de casos nuevos de cáncer en las distintas regiones y que el mayor número de casos se centran en los departamentos como Antioquia, Atlántico, Valle del Cauca, Cundinamarca, y Bogotá. Las estimaciones de incidencia denotan que la demanda de servicios varía de acuerdo con las características geográficas, y por esta razón, usualmente se consideraría que la oferta de servicios oncológicos debe darse acorde con la demanda local. Sin embargo, ya ha sido demostrado en el mundo que para garantizar adecuados desenlaces en salud, una razón de casos por IPS de 165 por año como se encontró en este estudio es muy baja teniendo en cuenta que los centros integrales de tratamiento como el Instituto Nacional de Cancerología atiende cerca de 7,000 casos nuevos por año; no obstante este hecho es particular por ser una institución referente que atiende pacientes de todo el país y no segmenta su oferta a regiones. ^4^. De la misma manera, países como el Reino Unido que cuenta con una incidencia de 273 (TAE por 100,000 años-persona) plantea que debe haber un centro integral de tratamiento por cada 2.5 a 3.6 millones de personas ^13^, concepto que llevado al contexto de Colombia significaría la tenencia de más o menos 15 centros integrales que ofrezcan una multitud de servicios oncológicos, lo cual no es la situación presente para el país.

En la última década la tendencia de la oferta de servicios de salud oncológicos en Colombia estuvo orientada a la habilitación de servicios del grupo de consultas de especialidades médicas oncológicas, con un segundo y tercer escalafón en los servicios de apoyo diagnóstico y complementación terapéutica y quirúrgicos; resultado de esta dinámica más de la mitad de los servicios oncológicos son de consulta externa, más del 65% de los prestadores que habilitan servicios de tratamiento para el cáncer inclinan su oferta a un solo tipo de servicio y solo el 6.5% de las IPS cuentan de forma integral con servicios de quimioterapia, radioterapia y cirugía oncológica. Este desbalance en la proporción de grupos de servicios y el bajo promedio de servicios habilitados por IPS sugieren la existencia de centros sanitarios que no integran las modalidades terapéuticas oncológicas básicas, lo cual dificulta la coordinación institucional e interpone barreras de acceso y en la calidad en la atención ^14^. 

Pese a que el presente artículo no es un análisis de suficiencia de servicios oncológicos por especialidad, pues incluye variables referentes a la capacidad productiva de los servicios, (infraestructura, talento humano, producción, tiempos y movimientos), el análisis si mostró que los servicios de ginecología y cirugía de mama y de tumores de tejidos blandos fueron las especialidades con más alta oferta en consulta, datos concordantes con los principales tipos de cáncer en mujeres (mama y cuello uterino). En el caso de ginecología oncológica su elevada oferta sí refleja la prioridad otorgada para brindar la atención de las patologías que trata la especialidad, de modo que con la oferta actual, cada servicio de consulta de ginecología oncológica habilitado atiende en promedio 30 casos nuevos de cáncer de cuello de útero al año, de los aproximadamente 5.000 casos diagnosticados; no obstante, la consulta de mama no necesariamente es habilitada para la atención de cáncer, por lo que las cifras no corresponden de forma tácita a la oferta para el tratamiento de patología oncológica. Además si se considera que de los 29,734 casos nuevos de cáncer anuales que se presentan en hombres ^6^, el cáncer de próstata es el más frecuente con alrededor de 9,000 casos, se evidenció que fue baja la oferta en la consulta externa para urología oncológica, pues en promedio un servicio de consulta para esta especialidad atiende 210 casos nuevos de cáncer de próstata al año, cifra diferente de la observada para la atención de mujeres. 

Adicional, el registro señaló que sumada en conjunto la oferta para las consultas de las especialidades oncológicas de dermatología, rehabilitación, oftalmología, medicina nuclear y consulta de cirugía pediátrica, no representa más del 5% de la oferta total del grupo de servicios de consulta externa. 

Todos los servicios del grupo quirúrgico fueron habilitados por IPS, de otro modo no era factible dado los criterios del estándar de interdependencia para la habilitación de servicios quirúrgicos según la Resolución 2003 de 2014, norma que reglamenta la habilitación de servicios de salud. Algunos servicios de radioterapia y medicina nuclear para apoyo diagnóstico y complementación terapéutica fueron habilitados por profesionales independientes, sin embargo, y teniendo las exigencias del estándar de talento humano de la norma de habilitación, esta es una condición no viable, dado que el funcionamiento de estos servicios implica varios profesionales y no podría ser ofertado por un profesional único independiente. Hecho que refleja discordancias en el sistema de información del registro de prestadores en el REPS. 

En la enfermedad oncológica avanzada, el alivio del dolor y los cuidados paliativos es la única opción realista de tratamiento que le permite mejorar la calidad de vida ^14^. La asistencia paliativa junto con otras disciplinas como la rehabilitación, la nutrición y la salud mental son áreas del cuidado fundamentales en todas las fases de la enfermedad y constituyen los servicios de soporte oncológico, tal como lo propone el *Modelo de cuidado del paciente con cáncer* ^4^; así la consulta de dolor y cuidado paliativo fue la tercera más ofertada y la de rehabilitación oncológica fue entre todas las consultas, la tercera menos ofertada, cifras que indican la necesidad de fortalecer estos servicios de soporte. Pese a que esta deducción está sujeta exclusivamente a la clasificación de los servicios en el registro de prestadores de servicios, no excluye que la rehabilitación esté incorporada durante la prestación, y los servicios estén inscritos como rehabilitación “general”. De igual manera, las consultas habilitadas de dolor y cuidado paliativo no son exclusivas en el tratamiento del cáncer, por lo que puede estar sobreestimada la oferta para esta especialidad para oncología. 

### Fortalezas y limitaciones

La disponibilidad de un sistema de información que permite de forma actualizada realizar la caracterización de la oferta de servicios de salud es una herramienta de gran valor para los actores del sistema de salud, incluidos prestadores, pacientes y tomadores de decisiones. Esta ventaja permite diferenciar el enfoque oncológico de algunas especialidades, de forma tal que la caracterización de estos servicios en particular resulta ser lo más próximo a la oferta real disponible para la atención del cáncer.

Sin embargo, la calidad de la información contenida en el registro de prestadores presenta algunas irregularidades. La asimetría en los datos es una limitante que puede explicarse por la singularidad en el proceso de inscripción de los prestadores en el registro. Una de las causas más señaladas es la posibilidad que tiene los prestadores de inscribir los servicios bajo códigos enmarcados en el denominado ‘’otros’’, razón por la cual muchas especialidades que prestan servicios de consulta externa y quirúrgicos habilitan sus servicios de forma no diferenciada y generan las divergencias relativas observadas entre la oferta de las subespecialidades oncológicas. Sumada a esta limitante, años atrás los servicios de radioterapia y medicina nuclear, tanto de consulta externa como de apoyo diagnóstico y complementación terapéutica, fueron inscritos bajo un mismo código de servicio lo que no permitió diferenciarlos según su finalidad clínica. Por lo tanto, es posible que estos servicios aún no hayan sido actualizados a los nuevos códigos de inscripción y se encuentren clasificados de forma errónea. De manera que, los datos presentados se deben tomar con cautela.

Finalmente, los datos suministrados por el REPS presentaron algunas falencias per se del proceso de inscripción y verificación de los servicios, y ello limita el análisis de variables que pueden aportar datos relevantes. Se requiere, entonces, de la actualización de la información de los servicios, por parte de los prestadores a fin de aproximarnos con mayor veracidad a la situación real de la suficiencia asistencial de los servicios oncológicos en Colombia.

## Conclusión

Colombia es un país con una amplia oferta de servicios oncológicos, extensa en todo el territorio nacional con núcleos de oferta identificados principalmente en las ciudades capitales del país. La fuerza de la oferta de servicios oncológicos se concentra en prestadores privados con una mínima participación de entidades públicas pertenecientes al estado colombiano. En general, la relación entre los grupos de servicios es asimétrica, siendo la mayoría de servicios oncológicos de consulta externa con pocos prestadores que ofertan de forma conjunta los servicios bases del tratamiento oncológico, lo cual refleja la fragmentación en la prestación, hecho que no beneficia al paciente. Por tanto, es necesario redefinir el concepto de servicio oncológico bajo el enfoque de atención integral y la importancia de habilitar unidades, centros integrales de tratamiento y otras formas de atención que garanticen calidad en la atención con accesibilidad, integralidad y continuidad. Aún no se conoce con certeza la capacidad de servicios oncológicos para las necesidades actuales y futuras del país.
